# Making prediabetes visible in primary health care: a qualitative study of health care professionals’ perspectives

**DOI:** 10.1186/s12875-023-02230-2

**Published:** 2023-12-13

**Authors:** Katri Harcke, Marit Graue, Timothy Charles Skinner, Christina B. Olsson, Nouha Saleh-Stattin

**Affiliations:** 1 Department of Neurobiology, Care Sciences and Society, Division of Family Medicine and Primary Care, Karolinska Institutet, Huddinge, Region Stockholm Sweden; 2 Academic Primary Health Care Centre, Solnavägen 1E Torsplan, plan 7, Stockholm, 11365 Region Stockholm Sweden; 3 Department of Health and Caring Sciences, Western Norway University of Applied Sciences, Bergen, Norway; 4 Institute of Psychology, University of Copenhagen, Copenhagen, Denmark; 5 La Trobe Rural Health School, La Trobe University, Bendigo, Australia; 6 Australian Centre for Behavioural Research in Diabetes, Melbourne, Australia; 7 Department of Neurobiology, Care Sciences and Society, Division of Physiotherapy, Karolinska Institutet, Huddinge, Region Stockholm Sweden

**Keywords:** Prediabetes, Primary health care, Primary prevention, Interprofessional collaboration

## Abstract

**Background:**

People with prediabetes are at high risk of developing type 2 diabetes and its complications, such as cardiovascular diseases and premature mortality. Primary prevention and health maintenance are therefore imperative. Evidence has shown that prediabetes can be prevented or delayed with behavioural change, mainly in eating habits and physical activity. Interventions that use a person-centered approach can lead to improvements in self-management, quality of life, and health outcomes. Nevertheless, there is a need for further research that engages healthcare professionals and people with prediabetes in constructing and implementing preventive programs. The purpose of this study is to explore and describe how healthcare professionals perceive prediabetes, the current challenges in its detection and treatment, and what is needed to improve quality of care.

**Methods:**

This qualitative study was conducted in Region Stockholm. A total of 26 primary health care professionals participated in individual interviews: 15 diabetes nurses and/or district nurses, five general practitioners, five dietitians, and one physiotherapist. Interview transcripts were analyzed with qualitative content analysis.

**Results:**

The analysis revealed two main themes that emphasize the need to make prediabetes more visible in primary health care. Despite the healthcare professionals’ engagement and their motivation to improve prediabetes care, ad hoc practices and the absence of clear screening guidelines and referral pathways made it harder to focus on primary prevention. Supporting professionals in implementing structured care for people with prediabetes might encourage more efficient interprofessional collaboration and contribute to better strategies for promoting behavioural change.

**Conclusions:**

Establishing prediabetes care guidelines, supporting health care professionals´ knowledge and skills in prediabetes care, and implementing interprofessional referral pathways are some steps to enhance prediabetes detection and care precedence in primary health care. These steps could lead to more preventive care and ensure patient safety and health care equity.

**Supplementary Information:**

The online version contains supplementary material available at 10.1186/s12875-023-02230-2.

## Background

Prediabetes is defined as impaired fasting glucose and/or impaired glucose tolerance. People with prediabetes are at high risk of developing type 2 diabetes and its complications, such as cardiovascular diseases and premature mortality [1]. Some of these complications can appear at the stage of prediabetes [1]. Primary prevention and health maintenance are therefore imperative.

Primary prevention is proactive action that mainly targets high-risk groups not yet affected by the condition [2]. According to the WHO, supporting health promotion and disease prevention is an important goal of primary health care [3]. The Swedish Health and Medical Care Act states that primary prevention is a fundamental responsibility of primary health care [4]. Other countries, such as the United Kingdom, have similar goals [5].

Studies have shown that by changing the eating and physical activity habits of people with prediabetes, lifestyle interventions can delay or prevent type 2 diabetes [6–8]. International organizations have therefore recommended lifestyle interventions as the primary approach to managing prediabetes [1, 9]. Some countries, such as the United Kingdom and the United States, have developed population-based diabetes prevention programs for people with prediabetes or other risk factors for type 2 diabetes [10, 11]. However, Sweden has no such programs. There is, therefore, a need to develop a lifestyle program that is scalable and sustainable in the Swedish healthcare context.

Current evidence suggests that interventions using a person-centred approach improve self-management, quality of life, and health outcomes by facilitating participation and engagement [12–14]. However, programs in the United Kingdom and the United States currently use a top-to-bottom approach, which means that the people who need the interventions were not involved in the design or implementation of the programs. Thus, although the interventions are effective, barriers such as lack of time and the complexity of transport may prevent participants from taking part, especially those who are most disadvantaged [15]. Other barriers related to the health care professionals were lack of knowledge and time to inform people about these programs [15].

Our research group has therefore started a project to co-design a model for delivering an intervention to prevent or delay type 2 diabetes in people with prediabetes. This study is part of the first phase of the project, gaining a better understanding of health care professionals’ needs and perspectives. It contributes to the limited knowledge about primary health care professionals’ perceptions of prediabetes care, and information needed to co-design a person-centred model for behavioural change. To date, there is no similar research that we could find that looks at primary care health professionals’ perspectives on the management of prediabetes in Sweden. However, a study from New Zealand [16] showed that prediabetes care was not prioritized because other core functions in primary health care took precedence. Other studies published similar results on prediabetes having low priority [17, 18] and a recent retrospective cohort study by Tseng et al. 2022 [19] found that rates of prediabetes clinical care activities are low and have not improved between 2016 and 2021. The next step will be to interview persons with prediabetes about their perception of prediabetes and its treatment.

One method for engaging users in designing health intervention programs is human-centred design, also called design thinking [20]. Human-centered design has been used successfully in health care innovation networks, medical device design, and public health. It is well-suited for co-designing an innovative new model for a lifestyle change intervention that people with prediabetes know or believe they can accomplish as well as feasible to implement in primary health care.

### Aims

To explore how health care professionals perceive prediabetes, the current challenges in its detection and treatment, and what is needed to improve quality of prediabetes care.

## Method

### Design and setting

In this qualitative study, data were collected in Region Stockholm primary health care between 2021 and 2022. There are around 230 primary health care centres in Region Stockholm of different sizes and not all have teams or work in teams. The teams, when existing often include a nurse and a general practitioner and they consult or refer patients to other professionals as the allied health care professionals (dietitians, physiotherapists and occupational therapists). The allied health care professionals are often employed at the rehabilitation units and are part of the primary health care system but are usually not located at primary health care centres [21].

### Participants

Diabetes nurses working at primary health care centres in Region Stockholm were contacted through the regional diabetes network and invited to participate [21]. Those who agreed were contacted by e-mail and asked if they could recommend colleagues (other nurses, physicians, dietitians, and physiotherapists) who work with people with prediabetes and would be willing to participate in this study. Thirteen diabetes nurses agreed and asked colleagues to participate, two district nurses, five general practitioners, and an allied health care professional. The remaining allied healthcare professionals were recruited through the dietitian network and physiotherapist network. A total of 26 health care professionals agreed to participate. Information about the study and researcher was supplied and a signed a consent form was obtained before the interview. Fifteen diabetes nurses and/or district nurses, five general practitioners, six allied health professionals (five dietitians and one physiotherapist) were interviewed. All the nurses and general practitioners worked at primary health care centres, as did two of the allied health care professionals. Four allied health care professionals worked at rehabilitation units. We chose diabetes nurses to gatekeep the recruitment as they are the health professionals who usually work with people with prediabetes, and they have the most contact with other health professionals who work with prediabetes across the primary health care service in Stockholm. The researcher/interviewer had a previous co-working relationship with six of the interviewees. These interviews were analysed together with the last author to account for any possible bias.

### Data collection

A semi-structured interview guide developed by the researchers based on the study aim and research questions was used (supp 1), because we could not find any similar studies or interview guides. Two pilot interviews were conducted to test the guide. Both were included in the study, as the interviews indicated no need for change. The interviews were digital, using Zoom or Teams meeting platforms, because of COVID-19. They were carried out by the first author (KH) in Swedish and were 20 to 45 min long, video and audio recorded, but only the audio recording was transcribed verbatim and analysed. All interviews were conducted individually by KH who had previous experience conducting similar interviews in a different project. Data saturation was discussed and attained.

### Data analysis

Interview transcripts were analysed with qualitative content analysis [22]. Two of the researchers (KH, NSS) conducted the initial analysis, thoroughly and repeatedly listening to and reading the transcripts to achieve in-depth understanding. This was followed by extracting the relevant parts to gain a clearer picture and identifying meaning units, which were further condensed and labelled with codes. The codes were then grouped into subthemes. This was initially done separately by KH and NSS, and then together in dialogue with each other. The researchers (KH and NSS) agreed in the final step on two main themes that developed from the subthemes. All the co-authors discussed the findings to bring different perspectives and bring nuance to the interpretation of the dataset. To demonstrate credibility and authenticity to the results as described by Lindgren, Graneheim & Lundman [22], quotations from the transcripts are provided in the Results section. They were translated to English by KH and checked by NSS. The coreq checklist [23] guided the reporting (supplement 2).

## Results

All fifteen nurses were women and had worked in primary health care between 1 and 20 years. Two general practitioners were men and three women. They had worked in primary health care between 1 and 12 years. Three of the allied health care professionals were men and three women. They had worked in primary health care between 1 and 6 years.

The analysis revealed two main themes that emphasize the need to make prediabetes more visible and provide preventive care at primary health care centres (Table 1). The first theme, ‘Adding prediabetes to the agenda’, is about the need to recognize the condition. The second, ‘Striving to find strategies to implement prediabetes care’, illuminates the potential to enhance care.

**Table 1:** Main themes and subthemes

Adding prediabetes to the agenda • *Invisible condition and ad hoc practices raise risk of serious consequences* • *General practitioners are the gatekeepers – they decide who to screen and treat* • *Delayed referral shifts the focus from primary prevention to disease treatment*	Striving to find strategies to implement prediabetes care • *Professionals need knowledge and support to deliver prediabetes care* • *The chain of care should be adjusted to improve resource allocation and promote collaboration* • *Professionals think that people with prediabetes face challenges in self-management*

### Adding prediabetes to the agenda

Acknowledging prediabetes and putting it on the agenda makes early detection and prevention possible. The participants emphasized that prediabetes is an important health issue that is not prioritized in primary health care. However, because prediabetes is now a diagnosis, the condition is more visible and taken more seriously than before. This theme evolved from the following subthemes:

*Invisible condition and ad hoc practices raise risk of serious consequences*, *General practitioners are the gatekeepers – they decide who to screen and treat*, and *Delayed referral shifts the focus from primary prevention to disease treatment.*

#### Invisible condition and ad hoc practices raise risk of serious consequences

Health care professionals described prediabetes as a common health problem that is invisible because there are no recognizable symptoms. They noted that lack of national guidelines and unclear regional primary health care guidelines and routines could lead to serious consequences for people with prediabetes.

I think if we have the guidelines as you say, it will work much better, because right now I feel it’s a bit, ah, people have the wrong diagnosis. There are quite many who have the wrong diagnosis. (General practitioner 1)

#### General practitioners are the gatekeepers – they decide who to screen and treat

In the absence of guidelines and routines, individual general practitioners decided who to screen and who to refer to nurses or allied health care professionals. Allied health care professionals often get referrals for persons with type 2 diabetes and very few for persons with prediabetes despite that they have both time and resources.

It’s the doctor who makes an assessment based on what we’ve discussed is a risk patient. It’s a little bit part of [the individual general practitioner’s] way of working… The others [General practitioners] don’t do that. (Nurse 1)

I probably meet mostly people who have just developed diabetes, rather than prediabetes. All too often I hear people who have kind of got diabetes like 10 years ago, but like this: ‘No, I’m seeing a dietitian for the first time now.’ Huh! It really should be in case of prediabetes, then it should be a dietitian right away. (Allied health care professional 3).

For the same reason, professionals’ individual interest in prediabetes and engagement could determine the routines and structure of care at the primary healthcare centres.

#### Delayed referral shifts the focus from primary prevention to Disease treatment

Detecting and treating prediabetes is one of the general practitioners’ responsibilities. However, general practitioners’ perceptions of and attitudes about their role varied, and this impacted prediabetes care.

Yeah, that you don’t take it seriously, yeah. You want a real diagnosis of diabetes to start treatment. Less than that, then you ignore it. Unfortunately, that’s how it happens. (General practitioner 5)

The nurses were explicit about the need to work with primary prevention of type 2 diabetes by prioritizing prediabetes. The allied health care professionals differed in their perceptions of their role in prediabetes care. They wanted to be involved earlier, that is before people developed type 2 diabetes.

### Striving to find strategies to implement prediabetes care

The participants emphasized that prediabetes care is suboptimal. They felt that they could do more to prevent type 2 diabetes and described things that could make a difference. This theme was based on three subthemes:

*Professionals need knowledge and support to deliver prediabetes care*, *The chain of care should be adjusted to improve resource allocation and promote collaboration*, and *Professionals think that people with prediabetes face challenges in self-management*.

#### Professionals need knowledge and support to deliver prediabetes care

The health care professionals indicated the importance of continual learning to find tools to provide person-centered prediabetes care.

I feel that I have to find strategies and ways together with patients. . I feel that I need support about psychology. In other words, I feel strongly that I would need a course in behavioural change. How do you help patients change their behaviour? (Nurse 15)

They also described a need for support from managers and colleagues.

It’s also a lot about this with the management and getting onboard, that you get the okay to work with this. As long as we don’t have that, we won’t get anywhere. (Nurse 1)

#### The chain of care should be adjusted to improve resource allocation and promote collaboration

General practitioners and nurses highlighted a lack of resources and time for prediabetes care. They noted that the person with prediabetes is often left on their own. Allied health care professionals, on the other hand, expressed the need for a better structure of prediabetes care.

We have the time. … We can offer qualified counselling consultations, but we don’t get any referrals. That’s my picture of primary care, that it works poorly. A patient doesn’t come to the right care provider, too. Thus, the chain of care simply doesn’t work. (Allied health care professional 1)

According to the allied healthcare professionals, teamwork and collaboration were inadequate, but they expressed some strategies to improve this collaboration.

There’s a lot you can do! To meet at various meetings or perhaps discuss various issues via joint meetings or in interprofessional collaborations – if I’m at a meeting with the general practitioners and that we have a common agenda. (Allied health care professional 2)

#### Professionals think that people with prediabetes face challenges in self-management

The health care professionals thought that people with prediabetes faced challenges in making changes in their daily lives, perhaps because they needed more time and knowledge. Another challenge could be a person’s reaction to their prediabetes diagnosis.

It’s everything from getting really scared to basically ignoring it: ‘Aha, at least it wasn’t real diabetes.’ (Nurse 10).

All three groups expressed the belief that people with prediabetes need concrete support that they can use in everyday life. This included support from primary health care and more support from family, relatives, and the community.

The health care professionals emphasized the need for continuous support to help people with prediabetes sustain behavioural change.

I think follow-up is very important, that you follow up with them and not just leave them with information the first time that you should exercise more and eat more healthily and stop smoking and every now and then leave them and have no follow-up for them. Then they’ll drop [out]. (General practitioner 5)

## Discussion

The main findings of this study indicated that primary care health professionals believe that effective management of prediabetes should be a priority for their services but feel it is not a priority at the primary health centres. However, despite the health care professionals’ engagement and their motivation to improve prediabetes care, several factors make it a challenge to achieve. Like lack of internal support (knowledge and skills) combined with lack of external support (managerial and structure) are some factors that contribute to not prioritizing prediabetes care.

For instance, the absence of clear screening and referral pathways reduces opportunities to carry out primary prevention, a fundamental responsibility of primary health care [4] and of the health care professions [24–26]. The primary health care system is failing to offer structured care for people with prediabetes, and health care professionals find themselves prioritizing treatment for the sickest rather than identifying and treating those at risk of getting sick.

In the present study, the general practitioners felt ambivalent about prioritizing prediabetes because it is a condition that does not require medical treatment until it becomes a disease (type 2 diabetes). Even in countries where standard care for prediabetes includes medical treatment, general practitioners can feel ambivalent about making a diagnosis and providing medical treatment to people who are not sick and do not have the symptoms of a disease [18, 27]. Because of their ambivalence, general practitioners in the current study found themselves in the position of gatekeepers, that is, of making ad hoc, individual decisions about prediabetes screening and treatment. Consequences include missed opportunities to identify people at risk and delay or prevent the development of type 2 diabetes and cardiovascular complications, as well as care inequity. On the other hand, nurses and allied health care professionals were clearer and more decisive about prediabetes screening and treatment.

The current study found that primary health care professionals often missed the opportunity to support people in self-managing their prediabetes through interprofessional collaboration. General practitioners’ ad hoc, individual decisions played a crucial role in whether other professionals had the chance to support people in self-management. Collaboration is of the utmost importance in supporting self-management [28]. By working in teams, tasks and responsibilities can be divided appropriately and according to the available resources. Having common treatment plans and goals, using the same language, and sending consistent messages to people with prediabetes helps ensure better outcomes [28]. The general practitioners, nurses, and allied health care professionals in the current study wanted to collaborate more closely to support people with prediabetes in self-management. This finding is consistent with the results of a survey of general practitioners’ perceptions of prediabetes [29]. The general practitioners in that study thought that expanding other team members’ roles could improve prediabetes identification and care.

Clinical practice guidelines are an important first step towards standardized, evidence-based practice and optimized care [30]. However, studies find that even when there are guidelines about detecting and treating prediabetes, lack of time, resources, and knowledge can make it hard to follow them [16, 29, 31]. This is in line with our results, as the health care professionals felt that they lacked skills and resources and needed continuing education about prediabetes and communication skills. Support from the primary health care centre manager is crucial in creating opportunities for health care professionals to improve their competence and knowledge about prediabetes care.

### Limitations and strengths

The study had some limitations. Interviews with the managers at the primary healthcare centres could have strengthened the findings by illuminating their perspectives on prediabetes care. Additionally, because of the COVID-19 pandemic, the interviews were conducted digitally. On the one hand, this facilitated participation. On the other, it reduced the ability to observe body language and other non-verbal cues and may have affected the dialogue.

The interviewer and first author, KH, is a district nurse who works clinically with prediabetes and diabetes care at a primary health care centre. Her insider perspective was both a strength and a limitation. It provided insight but could have affected her interpretation of the transcripts. It was therefore particularly important that the other authors provided insights from the perspectives of their different professions and backgrounds.

Because health care systems and other contextual factors vary by country, the findings may not be transferable to other settings. However, the overall challenges illuminated in the study are relevant in other contexts. Examples include the need for continuing education and support, the desire to work as an interprofessional team, and the need to make prediabetes more visible and provide preventive care.

## Conclusions

Establishing prediabetes care guidelines, supporting health care professionals´ knowledge and skills in prediabetes care, and implementing interprofessional referral pathways are some steps to enhance prediabetes detection and care precedence in primary health care. These steps could lead to more preventive care and ensure patient safety and health care equity.

### Electronic supplementary material

Below is the link to the electronic supplementary material.

Supplementary Material 1

Supplementary Material 2

## Data Availability

The data that support the findings (individual interview transcripts in Swedish) are not openly available because doing so would violate our Swedish Ethical Review Authority. Data are located in controlled access storage at the Karolinska Institutet and the codes, in English, are available from the corresponding author upon reasonable request.
