# Supplementary material for: Making prediabetes visible in primary health care: a qualitative study of health care professionals’ perspectives
Source: BMC Prim Care. 2023 Dec 13;24:266. doi: 10.1186/s12875-023-02230-2 (PMC10717089; doi:10.1186/s12875-023-02230-2)
Supplement: Supplementary file 1 — Supplementary Material 1 [file 12875_2023_2230_MOESM1_ESM.docx]

Supplement 1

**Interview guide**

Open questions to health care professionals working with people with prediabetes in primary health care:

- How do you view prediabetes? (How important is it to screen for prediabetes?)

- Can you tell us how you work or want to work with people with prediabetes to slow down or prevent the development of type 2 diabetes.

- (If they screen) Tell me, how do you do it? (What happens next… do you diagnose, do you have any guidelines that you follow?)

- What do you see as obstacles or opportunities in the work with prediabetes?

- Based on your experience, can you tell us how people who have been diagnosed with prediabetes react?

- What do you think people with prediabetes need for support to be able to take care of their prediabetes?

- What do you think are the obstacles or opportunities for people who have been diagnosed with prediabetes to make changes in their lifestyle / behavioural changes?
